# Influence of Estrus on Dairy Cow Milk Exosomal miRNAs and Their Role in Hormone Secretion by Granulosa Cells

**DOI:** 10.3390/ijms24119608

**Published:** 2023-06-01

**Authors:** Wenju Liu, Chao Du, Liangkang Nan, Chunfang Li, Haitong Wang, Yikai Fan, Ao Zhou, Shujun Zhang

**Affiliations:** 1 Key Laboratory of Agricultural Animal Genetics, Breeding and Reproduction of Ministry of Education, Huazhong Agricultural University, Wuhan 430070, China; 2 College of Life and Health Science, Anhui Science and Technology University, Fengyang 233100, China; 3 Laboratory of Genetic Breeding, Reproduction and Precision Livestock Farming, School of Animal Science and Nutritional Engineering, Wuhan Polytechnic University, Wuhan 430023, China

**Keywords:** exosome, dairy cow, milk, miRNAs, hormonal synthesis, gene regulation, granulosa cell

## Abstract

Estrus is crucial for cow fertility in modern dairy farms, but almost 50% of cows do not show the behavioral signs of estrus due to silent estrus and lack of suitable and high-accuracy methods to detect estrus. MiRNA and exosomes play essential roles in reproductive function and may be developed as novel biomarkers in estrus detection. Thus, we analyzed the miRNA expression patterns in milk exosomes during estrus and the effect of milk exosomes on hormone secretion in cultured bovine granulosa cells in vitro. We found that the number of exosomes and the exosome protein concentration in estrous cow milk were significantly lower than in non-estrous cow milk. Moreover, 133 differentially expressed exosomal miRNAs were identified in estrous cow milk vs. non-estrous cow milk. Functional enrichment analyses indicated that exosomal miRNAs were involved in reproduction and hormone-synthesis-related pathways, such as cholesterol metabolism, FoxO signaling pathway, Hippo signaling pathway, mTOR signaling pathway, steroid hormone biosynthesis, Wnt signaling pathway and GnRH signaling pathway. Consistent with the enrichment signaling pathways, exosomes derived from estrous and non-estrous cow milk both could promote the secretion of estradiol and progesterone in cultured bovine granulosa cells. Furthermore, genes related to hormonal synthesis (*CYP19A1*, *CYP11A1*, *HSD3B1* and *RUNX2*) were up-regulated after exosome treatment, while exosomes inhibited the expression of *StAR*. Moreover, estrous and non-estrous cow-milk-derived exosomes both could increase the expression of *bcl2* and decrease the expression of *p53*, and did not influence the expression of *caspase-3*. To our knowledge, this is the first study to investigate exosomal miRNA expression patterns during dairy cow estrus and the role of exosomes in hormone secretion by bovine granulosa cells. Our findings provide a theoretical basis for further investigating milk-derived exosomes and exosomal miRNA effects on ovary function and reproduction. Moreover, bovine milk exosomes may have effects on the ovaries of human consumers of pasteurized cow milk. These differential miRNAs might provide candidate biomarkers for the diagnosis of dairy cow estrus and will assist in developing new therapeutic targets for cow infertility.

## 1. Introduction

With the development of cow heritage improvement, milk production has significantly increased. Conversely, reduced reproductive performance has been reported, including an extended an estrus after partus, less pronounced estrous behavior and silent estruses [1,2,3], although artificial insemination has been widely used. Cow estrus is crucial for fertility and the profitability of modern dairy farms, and it affects milk production and the dairy farm economic benefit. Hence, monitoring cow estrus is important for successful artificial insemination and pregnancy. In spite of widely used artificial insemination, the pregnancy rate is still below 50% [4]. Reproductive hormones are the most viable option to induce estrus followed by artificial insemination [5]. However, this method still needs to be improved, and 5–30% of cows are not in estrus when they are inseminated [6]. Although many estrus-detection methods have been used to monitor dairy cow estrus, these methods have proven to be time-consuming, with higher cost and lower usefulness and effectiveness. Automated estrus-detection aids have been developed to take advantage of pressure-sensitive devices, pedometers, activity monitors, radiotelemetric devices, temperature detectors, neck-mounted activity tags and video systems [5,7,8], but these automated technologies require special equipment and management, resulting in increased financial investments.

Milk is abundant and readily available, and many researchers have paid more attention to monitor cow estrus by using the milk [2]. Moreover, collecting milk sample is noninvasive and does not require additional handling of live animals [9]. During estrus, the concentrations of fatty acids are greater in the urine of estrous cows than non-estrous cows [10]. Furthermore, the concentrations of some milk fatty acids change during estrus and non-estrus in lactating dairy cows; for example, the concentrations of acetic acid, valeric acid, caproic acid and myristoleic acid are greater in estrous compared to non-estrous cows [11]. Therefore, estrus could induce changes in the specific milk fatty acid profiles [12,13]. In addition, Du et al. [14] reported that estrus had a significant effect on milk production, including fat, protein, urea, total solids, and solid not fat, whose contents increased in estrous compared to non-estrous cows. Moreover, estrus also could change some of the wavenumbers in the water-absorption regions by mid-infrared spectroscopy detected on the days before and on the day of estrus. Therefore, changes in milk and milk components can be used as a potential means for detecting estrus expression.

Milk exosomes have attracted much attention by researchers and may be used as biomarkers for detection [15]. Exosomes are membrane vesicles that are derived from multiple cells and range in size from 30 to 150 nm in diameter. Moreover, exosomes contain functional components including proteins, microRNA and messenger RNA [16]. Importantly, membranes of exosomes can protect the cargo from degradation by extracellular proteases [17]. Evidence has shown that exosomes can be absorbed by the recipient cells and then act as regulators to alter the recipient cell function through the transfer of their cargo [18]. Recent studies have supported that exosomes play an important role in cell-to-cell communication, cellular trafficking, immune function and reproduction [17,19,20]. In addition, the cross talk between exosomal miRNAs and reproductive regulation is well established, including steroid hormone secretion [21,22] and embryo implantation and development [23,24]. However, to our knowledge, there is no related research on the difference in milk exosomes derived from estrous and non-estrous dairy cows. Therefore, the objective of this study was to investigate the miRNA expression profile and identify estrus-associated miRNAs in milk-derived exosomes from estrous and non-estrous dairy cows. Specifically, we explored the role of milk-derived exosomes in hormone secretion by bovine granulosa cells.

## 2. Results

### 2.1. Identification of Exosomes Isolated from Cow Milk

**Figure 1:**
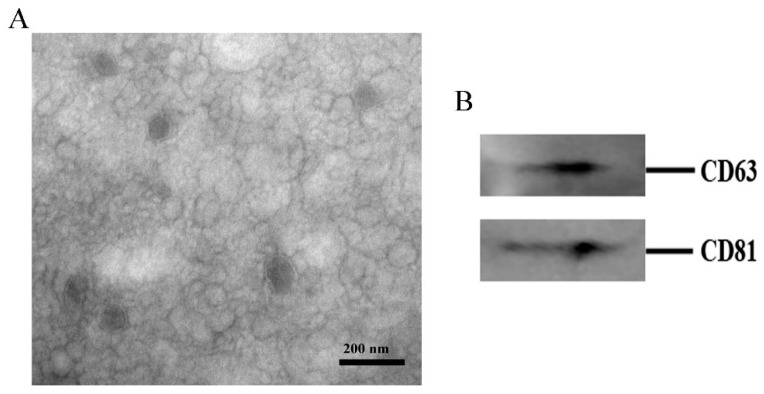
Identification of the exosomes isolated from cow milk after ultracentrifugation. (**A**) Transmission electron microscopic (TEM) image of exosomes in milk. (**B**) Western blot of known exosome markers (CD63 and CD81).

The morphology and size of exosomes extracted from cow milk were first observed by TEM. Cup-shaped, lipid-layer structures up to 200 nm in diameter were found (Figure 1A). In addition, the expression of exosomal markers was detected via Western blotting, and the results showed that the isolated milk exosomes abundantly contained the exosomal markers CD63 and CD81 (Figure 1B), suggesting that exosomes were successfully isolated from cow milk and could be used for further research. 

### 2.2. Characterization of Exosomes Derived from Estrous and Non-Estrous Cow Milk

**Figure 2:**
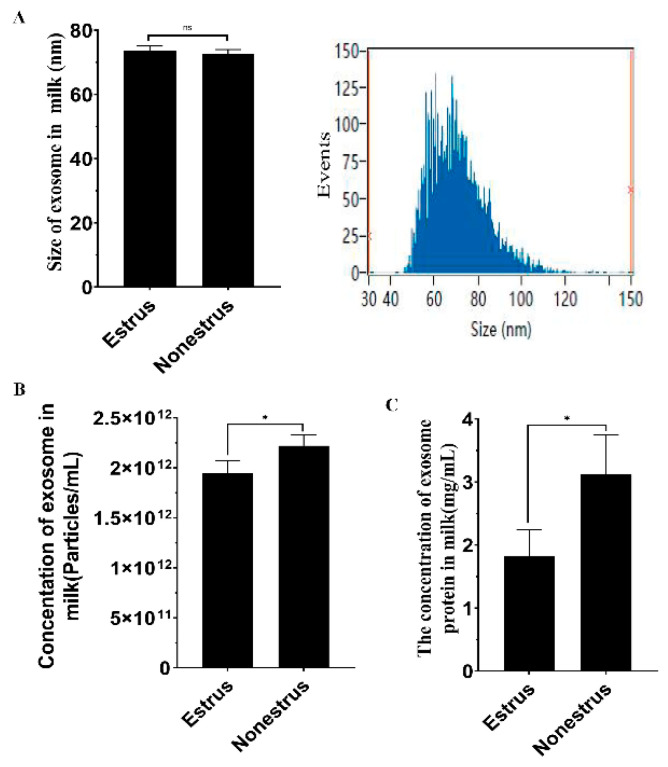
Characterization of exosomes derived from estrous and non-estrous cow milk. (**A**) Nano-flow cytometry analysis showing particle size distribution in exosomes derived from estrous and non-estrous cow milk. (**B**) Nano-flow cytometry showing the concentration of exosomes isolated from estrous and non-estrous cow milk. (**C**) Concentration of exosome proteins isolated from estrous and non-estrous cow milk. ns represents non-significant (*p* > 0.05), * *p* < 0.05. Data are presented as the mean ± standard deviation (SD).

To investigate the characteristics of the exosomes derived from estrous and non-estrous cow milk, nanoparticle size distribution was used to examine the size of the exosomes. We found that the mean size of estrous and non-estrous cow-milk-derived exosomes was 74.05 and 72.53 nm in diameter, respectively, and there was no significant difference between them (Figure 2A, *p* > 0.05). Nano-flow cytometry analysis indicated fewer exosomes and lower exosome protein concentration in estrous cow milk compared to non-estrous milk (Figure 2B,C, *p* < 0.05). These data indicated that estrus can influence exosome secretion and its function. 

### 2.3. Summary of Data Quality 

**Figure 3:**
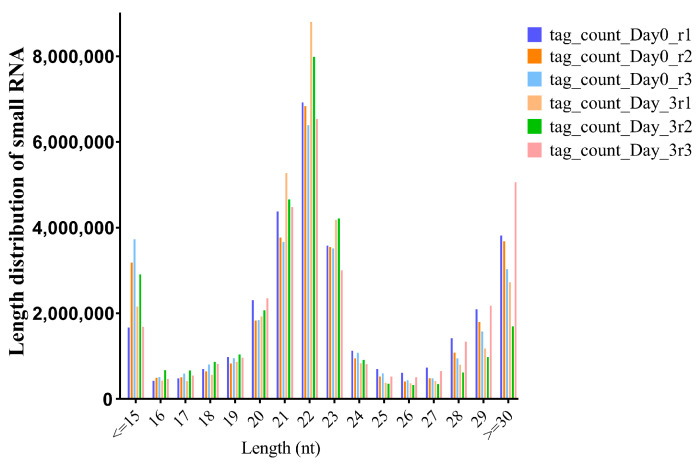
Length distribution of small RNA from BGISEQ-500 platform sequencing of milk exosome samples (nt = nucleotides). Day0 and Day –3 represent estrous and non-estrous milk exosome samples, respectively.

Exosomal miRNAs were found to serve as biomarkers for diagnosis. Herein, RNA-seq was preformed to reveal the function of exosomes in estrous and non-estrous cow milk. After processing the raw sequencing data, clean reads were obtained from estrous and non-estrous cow milk exosomes, and >91.26% of the reads were successfully coordinated to the *Bos taurus* reference genome (ARS-UCD1.2, Supplementary Table S1). Further analysis of identified miRNAs indicated that the length was mostly distributed on 21–23 nt. MiRNA with size length of 22 nt accounted for the highest number, followed by 21 nt and 23 nt (Figure 3, Supplementary Table S2).

### 2.4. Identification of Differentially Expressed miRNAs

**Figure 4:**
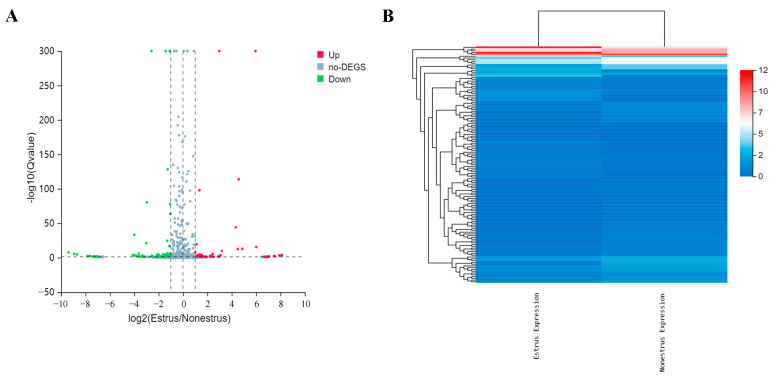
Identification of differential miRNAs in estrous and non-estrous cow milk exosomes. (**A**) Volcano plots of milk exosomal differentially expressed miRNAs between estrous and non-estrous cows. *X*-axis denotes fold change (log2); *Y*-axis refers to the q value (−log10). DEGS represents differentially expressed miRNAs. (**B**) Hierarchical clustering of differential miRNAs.

A total of 133 differentially expressed miRNAs were identified, including 70 known miRNAs and 63 novel miRNAs (Supplementary Table S3). Among them, 52 miRNAs were significantly upregulated in the exosomes derived from estrous compared with non-estrous cow milk, and 81 miRNAs were significantly downregulated (Figure 4A). In addition, a hierarchical clustering heatmap showed the expression pattern of differentially expressed miRNAs between the estrous and non-estrous cow milk exosomes (Figure 4B). 

### 2.5. miRNA Target Analysis

**Figure 5:**
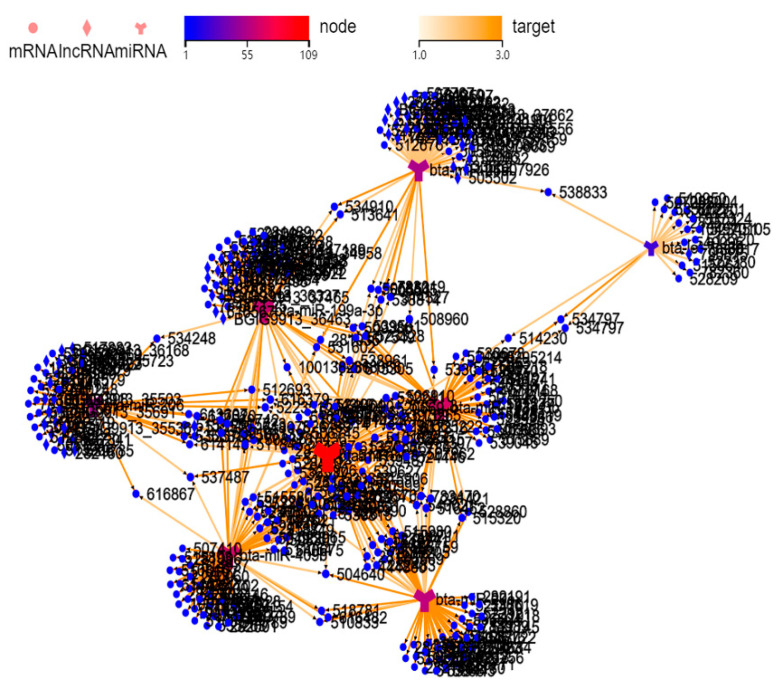
Prediction of miRNA-target interactions and construction of a miRNA–mRNA/lncRNA regulatory network.

To further investigate the interaction between miRNA and mRNA/lncRNA, we used databases, including RNAhybrid, miRanda and TargetScan, to predict target genes of miRNAs. According to the identified differentially expressed miRNAs, a miRNA–mRNA/lncRNA regulation network was built based on miRNA–mRNA/lncRNA interactions (Figure 5, Supplementary Table S4).

### 2.6. GO and KEGG Analysis of Differentially Expressed miRNA

**Figure 6:**
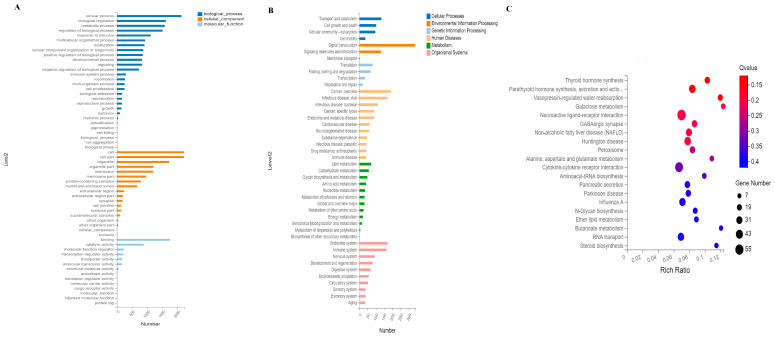
Gene ontology (GO) and KEGG enrichment analysis. (**A**) GO enrichment analysis of differentially expressed miRNAs. (**B**) KEGG enrichment analysis of differentially expressed miRNAs. (**C**) KEGG enrichment analysis of the top 20 differentially expressed miRNAs.

GO enrichment analysis of the target genes of differentially expressed miRNA was performed. The results showed that the target genes were mainly involved in biological processes, including cellular processes, biological regulation, metabolic processes, regulation of biological processes, developmental processes, reproduction, reproduction processes and signaling (Figure 6A, Supplementary Table S5). Moreover, the target genes related to the cellular components were mainly associated with cells, cell parts, organelles, membranes, protein-containing complexes and membrane-enclosed lumens. The target genes involved in molecular functions mainly included binding, catalytic activity, molecular function regulator, transcription regulator activity and transporter activity. The differentially expressed miRNA may be beneficial for exosomes involved in regulating communication. 

According to KEGG enrichment analyses, signal transduction, signaling molecules and interaction, transport and catabolism, lipid metabolism, endocrine system, development and regeneration were closely connected to the exosome function and found to contain higher numbers of the target miRNA genes (Figure 6B, Supplementary Table S6). The top 20 pathways were identified based on the KEGG pathway analysis. Thyroid hormone synthesis, parathyroid hormone synthesis and secretion, neuroactive ligand–receptor interaction and steroid biosynthesis were among these pathways (Figure 6C).

### 2.7. Validation of miRNA Expression by qPCR

**Figure 7:**
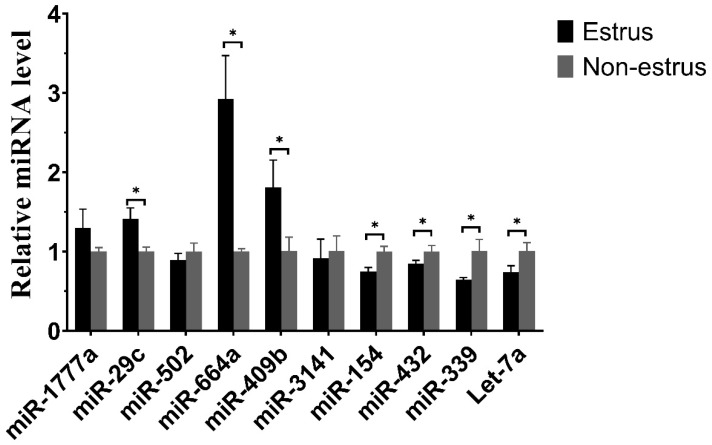
Quantitative verification results of miRNA. The relative expressions of 10 miRNAs in estrous and non-estrous milk exosome samples were verified. * *p* < 0.05.

To evaluate the accuracy of sequencing, we selected ten differentially expressed miRNAs to verify the RNA-seq sequencing data by qPCR (Figure 7). The results of qPCR were similar to the miRNA-sequencing in estrous and non-estrous cow-milk-derived exosomes, which supported the reliability of the miRNA-sequencing.

### 2.8. The Effect of Exosomes on Hormone Secretion and Endocrine-Related Gene Expression

**Figure 8:**
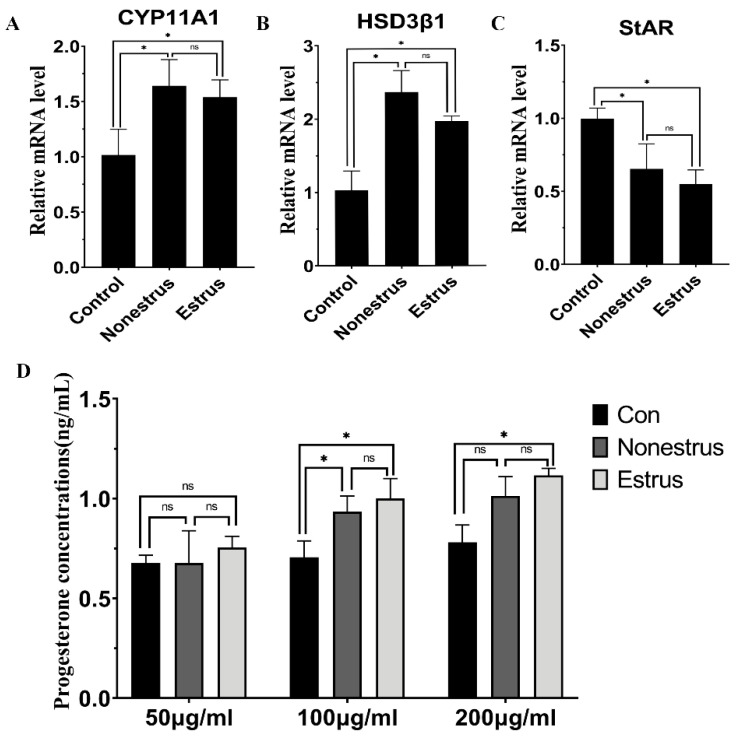
Effects of milk exosome supplementation on progesterone secretion and endocrine-related gene expression (*CYP11A1*, *StAR* and *HSD3β1*). The mRNA levels of *CYP11A1* (**A**), *HSD3β1* (**B**) and *StAR* (**C**) were examined by real-time PCR in granulosa cells at 24 h after exosome supplementation derived from estrous and non-estrous cow milk. The quantity of mRNA was normalized to that of *β-actin*. Abundance of progesterone (**D**) was measured at 24 h in granulosa cell medium after exosome supplementation derived from estrous and non-estrous cow milk. Statistical differences were evaluated using one-way ANOVA. ns represents non-significant (*p* > 0.05), * *p* < 0.05. The experiment was repeated three times independently.

**Figure 9:**
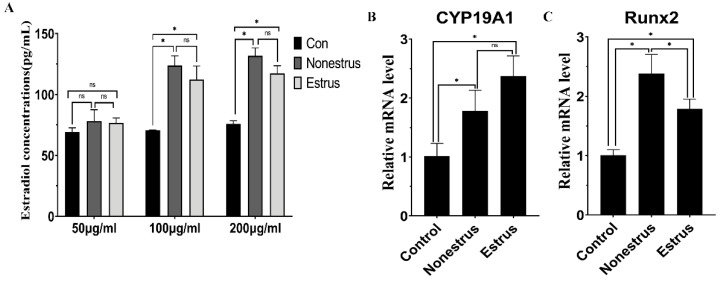
Effects of milk exosome supplementation on estradiol secretion and endocrine related gene expression (*CYP19A1* and *RUNX2*). Abundance of estradiol (**A**) was measured at 24 h in granulosa cells medium after exosome supplementation derived from estrous and non-estrous cow milk. The mRNA levels of *CYP19A1* (**B**) and *RUNX2* (**C**) were examined by real-time PCR in granulosa cells at 24 h after exosome supplementation derived from estrous and non-estrous cow milk. The quantity of mRNA was normalized to that of *β-actin*. Statistical differences were evaluated using one-way ANOVA. ns represents non-significant (*p* > 0.05), * *p* < 0.05. The experiment was repeated three times independently.

The level of estradiol and progesterone was measured 24 h after exosome treatment in bovine granulosa cells. The results indicated 100 and 200 μg/mL exosomes derived from estrous and non-estrous cow milk both could promote the production of progesterone and estradiol compared to control (*p* < 0.05), and there was no significant difference between estrous and non-estrous cow milk exosomes (*p* > 0.05, Supplementary Table S7). In addition, there was no significant difference between 100 and 200 μg/mL exosomes in promoting the secretion of estradiol and progesterone in granulosa cells (Figure 8D and Figure 9A, *p* > 0.05), suggesting that milk-derived exosomes can induce the production of estradiol and progesterone. 

To further confirm the effects of milk-derived exosomes on endocrine secretion, we investigated the expression of several hormone-related genes (*StAR*, *CYP19A1*, *CYP11A1*, *RUNX2* and *HSD3β1*) by real-time PCR using 100 μg/mL exosomes (Supplementary Table S7). The results showed that 100 μg/mL exosomes derived from estrous and non-estrous cow milk significantly upregulated the expression of *CYP19A1* and *CYP11A1*, which encode the rate-limiting step for estradiol and progesterone synthesis, respectively (Figure 8, *p* < 0.05). Moreover, the expression of *RUNX2* and *HSD3β1* involved in estradiol and progesterone synthesis was significantly increased after exosome treatment, respectively (Figure 8 and Figure 9, *p* < 0.05). The expression of *StAR*, an important regulator of progesterone synthesis, was significantly inhibited after exosome treatment (Figure 8C, *p* < 0.05). Furthermore, there was no significant difference in the expression of *StAR*, *CYP19A1*, *CYP11A1* and *HSD3B1* between estrous and non-estrous cow milk exosomes (Figure 8 and Figure 9, *p* > 0.05). However, the expression of *RUNX2* was significantly higher in exosomes derived from non-estrous cow milk than in exosomes derived from estrous cow milk (Figure 9C, *p* < 0.05). 

### 2.9. The Effect of Exosomes on Apoptosis-Related Gene Expression

**Figure 10:**
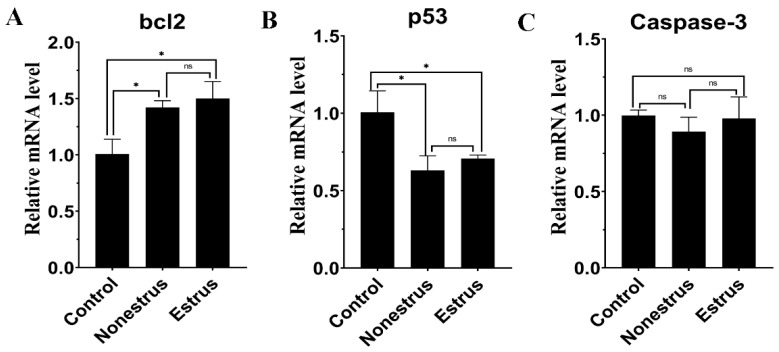
Effects of milk exosome supplementation on apoptosis-related gene expression (*bcl2*, *p53* and *caspase-3*). The mRNA levels of *bcl2* (**A**), *p53* (**B**) and *caspase-3* (**C**) were examined by real-time PCR in granulosa cells at 24 h after exosome supplementation derived from estrous and non-estrous cow milk. The quantity of mRNA was normalized to that of β-actin. Statistical differences were evaluated using one-way ANOVA. ns represents non-significant (*p* > 0.05), * *p* < 0.05. The experiment was repeated three times.

To further reveal the effects of exosomes on bovine granulosa cell apoptosis, the expression of *bcl2*, *caspase-3* and *p53* were measured after exosome treatment. Estrous and non-estrous cow milk exosomes both could promote the expression of *bcl2* (Figure 10, *p* < 0.05), while inhibiting the expression of *p53* (Figure 10, *p* < 0.05, Supplementary Table S7), and there was no difference between the estrous and non-estrous cow milk exosomes (Figure 10, *p* > 0.05). In addition, exosomes derived from estrous and non-estrous cow milk did not affect the expression of *caspase-3* (Figure 10, *p* > 0.05). Therefore, milk exosomes could inhibit bovine granulosa cell apoptosis.

## 3. Discussion

Estrus is important for optimizing the reproductive performance of dairy cows and consequently improve farm profitability [25], but about 50% of cows ovulate without expressing behavioral signs of estrus. Although there are many developed methods to monitor cow estrus, how to use milk to detect estrus in cows is still a subject worthy of consideration. Cow milk is a widely available source and contains many biomolecules, including cytokines, chemokines, hormones and exosomes [15,26], and milk exosomes play a role in reproduction. However, related research on how estrus affects the milk exosome secretion is still lacking, especially exosomal miRNA expression and the role of milk exosomes in the secretion of estradiol and progesterone in cultured bovine granulosa cells. Therefore, in the present research, we investigated the difference in milk exosomal miRNA expression between estrous and non-estrous dairy cows. We also explored the effect of milk exosomes in regulating hormone secretion and related gene expression in bovine granulosa cells. The results showed that estrus can indeed affect the milk exosomal miRNA expression and the concentration of exosomes derived from milk, and exosomes could influence the secretion of estradiol and progesterone as well as the related gene expression. The findings of this research provide a theoretical basis to further evaluate the role of milk exosomes and exosomal miRNAs in cow estrus.

Milk contains a variety of nutrients and bioactive agents, which are beneficial for consumers [27]. How to use milk to monitor the state of cow estrus is still worthy of investigation. Indeed, numerous studies have assessed the relationship between the estrous cycle and the consumption of milk and milk physical properties, and milk profiles could potentially be taken into account for monitoring cows showing estrus [12]. Changes in milk components, including urea content, somatic cell score, freezing point, pH and homogenization index, indicate variation associated with the hormonal and behavioral changes of cows undergoing estrus [13]. There are greater concentrations of fatty acids in milk on the day of estrus compared days of non-estrus [10,11]. In addition to the above milk components, exosomes and their miRNAs are emerging as novel functional components of milk [15,28]. To our knowledge, this is the first investigation where differences in milk exosomes on the days before and on the day of estrus have been reported. In the present research, we found that the number of exosomes and protein concentration were significantly lower in estrous cow milk than in non-estrous cow milk. Lopez et al. reported less time resting and eating, more time walking and a reduction in milk yield in cows standing during estrus [29]. Therefore, estrus affects physical measures and milk yield, which may cause changes in exosome number and protein concentration derived from milk. 

Estrus in high-producing cows is negatively associated with milk production [30]. Hence, reproductive efficiency is a particular concern, making estrus detection a priority [6]. Numerous studies have evaluated methods to detect estrus expression, including milk progesterone, milk profiles, heat mount detectors, activity monitors and wavenumbers in the water-absorption regions of milk [12,14,31,32]. However, these methods need special equipment and management, and about 20% of estrus expression is still not detected; thus, the accuracy needs to improve [31]. There is growing interest in exploring methods for estrus detection. The change in miRNAs has been investigated in follicles and plasma during estrus. Ioannidis & Donadeu [33] revealed the dynamic nature of plasma miRNAs during the estrous cycle and provide evidence of the feasibility of using circulating miRNAs as biomarkers of reproductive function in livestock in the future. The ovarian stroma and follicles exhibit differential expression miRNAs during estrus in goats [34,35]. Moreover, Gad et al. [36] revealed that heifers with divergent responses to ovarian superstimulation exhibited differential abundance of plasma extracellular vesicle miRNAs, which may be used as a potential biomarker to predict individual animal responses. Previous research on the characteristics of miRNAs mainly focused on ovaries, follicles and blood during estrus. Exosomal miRNAs have been widely investigated and considered as potential biomarkers for indicating mammals under normal or pathological conditions. In the present study, potential miRNA biomarkers of the response to estrus were identified in cow milk exosomes. We found that estrus could induce the differential expression of exosomal miRNAs, and 133 differentially expressed miRNAs were identified between the estrous and non-estrous cow milk exosomes. Among them, bta-let-7a-3p, bta-miR-26a, bta-miR-199a-3p, bta-miR-138, bta-miR-193a-3p and bta-miR-383 were involved in reproduction regulation. Let-7 family and miR-26a are considered the most abundant miRNAs in ovaries and follicles with a potential role in ovarian function [37,38]. miR-383 has stimulatory effects on estradiol production by mouse granulosa cells [39]. Consistent with the present study, plasma extracellular vesicle miRNA profiles show that miR-199a-3p is commonly up-regulated after superstimulation in both high- and low-responding heifers. Similarly, the expression of miR-199a-3p is increased in dominant follicles compared to subordinate follicles on day 7 of the estrous cycle in cattle [40]. Furthermore, miRNA contents of small follicular extracellular vesicles are modified depending on the estrous cycle stage and are associated with follicular P4 concentration [22]. The present results give more insights into potential biomarkers to predict individual animal responses to estrus. However, the mechanism of how estrus affects the expression of exosomal miRNAs in milk is still unknown.

Although milk exosomes are mainly secreted by the mammary glands [41], there are no known published studies related to the source of milk exosomes. The mammary blood flow volume is huge, about 30 L/min for the whole udder, while producing about 31.2 kg/d during lactation in dairy cows [42]. In addition, exosomes have been identified in blood, and the number of exosomes is about 3.5 × 10^10^ [23]. In the present study, the number of exosomes derived from milk (about 2 × 10^12^) is significantly higher than the exosomes in blood. This higher concentration of exosomes in milk may be due to circulating exosomes existing in the huge mammary blood flow volume. It has been shown that blood circulating exosome concentrations and abundance of exosomal miRNAs change during pregnancy [23,43] and ovarian superstimulation [36], respectively. 

Estradiol and progesterone are the main steroid hormones secreted by granulosa cells and play an important role in regulating follicle development [44]. The level of estradiol and progesterone in the follicle also reflects the stage of follicle development; for instance, higher estradiol concentrations were found in dominant follicles compared with those destined for atresia [45,46], and the greatest progesterone concentration was found in the largest follicles and related to the size of the follicles [47]. Moreover, the decreased duration of estrus has been related to the reduced systemic concentrations of estradiol and progesterone in high-producing lactating dairy cows [48]. Therefore, the hormones secreted by granulosa cells are closely related to follicle development and ovulatory capacity [44,49]. In the present research, we found that exosomes derived from estrous and non-estrous dairy cow milk both could induce the production of estradiol and progesterone in bovine granulosa cells. In addition, exosome treatment altering the granulosa cell transcriptome showed that the main differential genes affecting estradiol and progesterone synthesis were *CYP19A1*, *CYP11A1*, *HSD3β1* and *RUNX2*. Consistent with the present results, Yuan et al. [21] found that exosomes derived from follicular fluid promote progesterone synthesis as well as *CYP11A1* and *HSD3β1* genes in porcine granulosa cells. Curiously, contrary to promoting progesterone production, the expression of *StAR* was significantly inhibited in granulosa cells by exosome treatment both in estrous and non-estrous cow milk. Consistent with our study, the expression of *StAR* has a decreasing expression pattern, despite an increase in progesterone level [22,50,51,52]. A possible reason is that the high concentration of progesterone secreted by granulosa cells acts as a negative feedback regulator of *StAR* levels [22,51,52,53]. The main reason is that *StAR* may be inhibited from translocating cholesterol from outer to the inner mitochondrial membrane, with a decreasing expression pattern as a rapid response to higher progesterone synthesis [51,52,53]. Hung et al. [54] also reported similar results that follicular fluid exosomes promoted bovine granulosa cell proliferation. Similarly, extracellular vesicles obtained from small follicles are associated with a large number of upregulated genes that modulate biological processes involved in reproduction in cumulus cells [22]. Consistent with promoting the secretion of steroid hormones, milk exosomal miRNA KEGG analysis indicated that they were involved in cholesterol metabolism, FoxO signaling pathway, Hippo signaling pathway, MAPK signaling pathway, mTOR signaling pathway, PI3K-Akt signaling pathway, steroid hormone biosynthesis, TGF-beta signaling pathway, Wnt signaling pathway and GnRH signaling pathway, which are relevant and important for follicle development and oocyte maturation by regulating the biological processes, for instance, proliferation, cell differentiation and steroid hormone biosynthesis [21,22,49,55,56,57,58,59]. Thus, these results suggest that exosomes derived from milk could modulate estradiol and progesterone synthesis in cultured granulosa cells, and then impact the ovary functions, especially follicular development and oocyte maturation. However, the detailed mechanism is still unknown and requires further study in the future.

In addition to the estradiol and progesterone mentioned above, the stage of granulosa cells is also important and dictates follicular development and oocyte maturation [44]. Folliculogenesis is a dynamic process, from follicles to primary follicles, subsequently into secondary follicles and eventually reaching the preovulatory stage, during which 99% of follicles undergo atresia [60,61]. Researches have shown that follicular atresia can be initiated by granulosa cell apoptosis [60,62]. In the present study, we found that exosomes derived from estrous and non-estrous cow milk both could promote *bcl2* and inhibit *p53* gene expression. It is well known that *bcl2* and *p53* are involved in inhibiting or inducing cell apoptosis, respectively [23,63]. Moreover, estradiol and progesterone can also inhibit granulosa cell apoptosis [64,65]. A similar regulatory relationship is reported in porcine granulosa cells and intestinal epithelial cells, wherein follicular fluid exosomes and yak-milk-derived exosomes may inhibit cell apoptosis via the involvement of *bcl2* and *p53* [21,66,67]. Therefore, milk exosomes regulate granulosa cell function not only through promoting estradiol and progesterone secretion but also by inhibiting granulosa cell apoptosis. 

One of the major characteristics is that the synthesis of estradiol by granulosa cells is related to follicle development, oocyte maturation, ovulation and inhibiting granulosa cell apoptosis [64,68]. Interestingly, the present results showed that milk-derived exosomes could promote the secretion of estradiol and progesterone in bovine granulosa cells. However, it is still worth studying whether milk exosomes regulate the hormone secretion of human granulosa cells due to increasing cow milk consumption. Abundant milk miRNAs are packaged into exosomes [41], and these miRNAs may then be involved in regulating the granulosa cell function. Accumulated evidence has shown that miRNAs play an essential role in regulating estradiol synthesis in granulosa cells. miR-383, miR-133b and miR-132 enhance estradiol release from mouse ovarian granulosa cells [39,69,70]. miR-21, miR-20b and miR-31 repress their target gene expression in porcine granulosa cells to potentially promote estradiol production [71,72]. Moreover, miR-21 carried by human mesenchymal stem-cell-derived exosomes ultimately promotes estrogen secretion in ovarian granulosa cells (KGN and SVOG cells) [73]. Follicular fluid exosome-carried miR-31-5p promotes the proliferation of granulosa cells and progesterone synthesis in porcine granulosa cells [21]. Notably, milk exosomes reach the systemic circulation and accumulate in tissues following suckling, oral gavage and intravenous administration in mice, pigs and humans [74,75]. In addition, bovine milk exosomes and their miRNA cargo are bioavailable and accumulate in the placenta and embryo in mice. Of importance, bovine milk exosome-dependent changes in gene expression appear to promote embryonic growth and survival [76]. Therefore, bovine milk exosomes may have effects on the ovaries of human consumers of pasteurized cow´s milk. However, the mechanisms of the bovine milk exosomes how to regulate the reproduction function require further study. Although milk exosomes exhibit beneficial effects on granulosa cells, there is no evidence that milk exosomes reach the maternal circulation. The structure of the blood–milk barrier and tight junctions in the entire mammary gland might be involved in preventing an unhindered exchange from mammary gland to blood [77]. However, this needs to be further investigated in the future.

## 4. Materials and Methods

### 4.1. Milk Samples

Milk samples (n = 10) were collected from a commercial dairy farm in Hebei province of China. Lactating Holstein dairy cows with 1 parity were used in the present study. Feeding and management of cows were performed as previously described [14]. In brief, the cows were provided with a total mixed ration at 08:00, 16:00, and 24:00 h. Moreover, the cows were milked in a milk carousel with 80 milk stalls at 07:00, 15:00, and 23:00 h. After 21 days post-partum, the cows were synchronized with a Presynch-Ovsynch protocol as follows: prostaglandin F2α (PGF2α, Sansheng Biological Technology Co., Ltd. Zhejiang, China); 14 days later, PGF2α; 7 days later, gonadotropin-releasing hormone (GnRH, Sansheng Biological Technology Co., Ltd. Zhejiang, China); 7 days later, PGF2α; 3 days later, GnRH; 7 days later, GnRH; 7 days later, PGF2α; 24 h later, PGF2α; 32 h later, GnRH; and 14 h later, timed artificial insemination. Additionally, the day of timed artificial insemination was considered as day 0, and the pregnancy diagnosis was performed by an experienced veterinarian using both rectal palpation and ultrasound examination 5 weeks after timed artificial insemination. Milk samples were collected on day –3 and day 0 from the same cow. The cow is considered estrous on day 0 when the pregnancy diagnosis is positive, and non-estrous on day –3 before artificial insemination. About 40 mL of milk was sampled during the morning milking. Samples were immediately sent back to the laboratory on dry ice and kept at −80 °C until use.

### 4.2. Exosome Preparation

Milk collected from estrous (n = 5) and non-estrous (n = 5) cows was used. The exosome was isolated as described previously [66,78,79]. Briefly, 15 mL milk was centrifuged at 5000× *g* for 30 min at 4 °C to remove fat, large debris and cells. The supernatant was centrifuged again at 12,000× *g* for 30 min to eliminate residual fat and cell debris. Then, an equal volume of 0.25 M EDTA (Sigma, St Louis, MO, USA, pH 7) was added to the defatted supernatant, which was then incubated for 15 min on ice to precipitate casein and exosomes coated with casein as described by Kusuma et al. [80]. The clear supernatant was then passed through 0.45 μm and 0.22 μm filters to remove residual cell debris and ultracentrifuged at 120,000× *g* for 90 min at 4 °C (Beckman, SW41T rotor, Brea, CA, USA). The pelleted exosomes were resuspended in PBS and then ultracentrifuged at 120,000× *g* for 90 min at 4 °C for washing. Finally, the exosomes were resuspended in PBS and then stored at −80 °C until use.

### 4.3. Transmission Electron Microscopy

The exosomes were placed on a 200 mesh copper grid for 2 min. Subsequently, excess liquid was removed with filter paper, and the grid was negatively stained with 2% uranyl acetate (Beijing Zhongjingkeyi Technology Co., Ltd., Beijing, China) for 1 min. The grid was washed by moving it onto several drops of double-distilled water. The grid was air dried and then observed by a transmission electron microscope at 100 kv (H-7650, HITACHI, Tokyo, Japan).

### 4.4. Exosome Protein Quantification

The total protein concentrations of exosomes collected from estrous (n = 5) and non-estrous cow milk (n = 5) were measured with the BCA Protein Assay Kit using bovine serum albumin (BSA) as the standard according to the manufacturer’s instructions (Beyotime, Shanghai, China). All samples were tested 3 times. 

### 4.5. Exosome Characterization and Quantification

The concentration and size distribution of exosomes derived from estrous (n = 5) and non-estrous cow milk (n = 5) were analyzed using nFCM (Nano-flow cytometry, Xiamen, China) according to reported protocols [81,82]. Briefly, two single-photon counting avalanche photodiodes (APDs) were used for the simultaneous detection of side scatter (SSC) and fluorescence of individual particles. The instrument was calibrated for particle concentration using 200 nm PE and AF488 fluorophore conjugated polystyrene beads and for size distribution using Silica Nanosphere Cocktail (Cat. S16M-Exo, NanoFCM Inc., Xiamen, China). Any particles that passed by the detector during a 1 min interval were recorded in each test. All samples were diluted to attain a particle count within the optimal range of 2000–12,000/min. Using the calibration curve, the flow rate and side scattering intensity were converted into corresponding vesicle concentration and size on the NanoFCM software (NanoFCM Profession V1.0).

### 4.6. Western Blot Analysis 

The total protein extracted from exosomes was loaded onto gels and separated by 12% polyacrylamide gel electrophoresis, and then transferred to polyvinylidene fluoride membrane (Millipore, Bedford, MA, USA). First, the blots were blocked and incubated overnight with primary mouse monoclonal antibodies (CD63, sc-5275 and CD81, sc-23962, Santa Cruz, Dallas, TX, USA) at 4 °C. Subsequently, the blots were incubated with HRP-labeled anti-mouse secondary antibody (SC-2005, 1:5000; Santa Cruz, Dallas, TX, USA). Finally, a ClarityWestern ECL kit was used for detection (Bio-Rad Laboratories, Hercules, CA, USA), and the bands were exposed using a ChemiDocXRS chemiluminescent imaging system (Bio-Rad, Hercules, CA, USA).

### 4.7. Small RNA Sequencing

Total RNA was isolated from milk exosomes derived from estrous (n = 3) and non-estrous cow milk (n = 3) using QIAzol Lysis Reagent (QIAGEN, Valencia, CA, USA). MiRNA regions of 18–30 nt were isolated and purified using a 15% urea PAGE gel. Subsequently, these purified miRNAs were combined with 3′ Adapter and 5′ Adapter. Then, the adapter-ligated miRNA underwent a reverse transcript reaction using SuperScript II Reverse Transcriptase (Invitrogen, Carlsbad, CA, USA), followed by 15 cycles of PCR amplification to enrich the cDNA fragments. The second size selection was carried out, and 100–120 bp fragments were selected from the gel and purified using the QIAquick Gel Extraction Kit (QIAGEN, Valencia, CA, USA). The distribution of the fragment sizes obtained was checked by an Agilent 2100 bioanalyzer (Thermo Fisher Scientific, Waltham, MA, USA). Single-stranded PCR products were produced via denaturation, which gave the final miRNA library. miRNA profiling was accomplished using the BGISEQ-500 platform (BGI, Wuhan, China).

### 4.8. MiRNA Analysis

Clean tags were obtained by processing the raw sequencing data as follows: removing low-quality tags, tags with 5′ primer contaminants and without 3′ primer; tags without insertions, tags with poly A and tags shorter than 15 nt. After filtering, the clean tags were mapped to the *Bos taurus* reference genome (ARS-UCD1.2) and other sRNA databases including miRbase, siRNA, piRNA and snoRNA with Bowtie2 [83]. The miRNA expression level was calculated by counting absolute numbers of molecules using unique molecular identifiers [84]. Differential expression analysis was performed using the DESeq [85] on the Dr. Tom Multi-omics Data Mining System (https://biosys.bgi.com (accessed on 22 November 2021). Q value ≤ 0.05 and |log2FC| > 1 was used to consider the significance of expression differences. For target gene prediction, we applied RNAhybrid [86], miRanda [87] and TargetScan [88] to predict target genes of miRNAs. For annotation, all target genes were aligned against the Kyoto Encyclopedia of Genes (KEGG) and Gene Ontology database and performed using phyper, a function of R on the Dr. Tom Multi-omics Data Mining System (https://biosys.bgi.com (accessed on 22 November 2021). A *p*-value ≤ 0.05 was defined as indicating significantly enriched terms.

### 4.9. Bovine Granulosa Cell Culture and Treatment with Exosomes

Granulosa cell isolation followed our previously described protocol [89,90,91]. Bovine ovaries were obtained from the local abattoir and sent back to the laboratory in a thermos cup. About 80 bovine ovaries were collected by washing three times using 70% alcohol, and then the ovaries were washed three times in sterile 0.9% NaCl to remove alcohol. The follicular fluid was obtained from follicles with a diameter of 5–8 mm and was centrifugated at 1500 rpm for 5 min. The collected cell pellets were digested by 0.25% trypsin with 0.025% EDTA (Gibco, Grand Island, NY, USA) for 5 min. After being digested, the cell pellets were centrifugated again and dispersed in Dulbecco’s Modified Eagle Medium (DMEM) (Gibco, Grand Island, NY, USA) supplemented with 10% fetal bovine serum (FBS; Hyclone, UT, USA) and antibiotics including streptomycin (50 µg/mL), penicillin (50 IU/mL) (Pen-Strep, Invitrogen, Carlsbad, CA, USA) and plasmocin (25 µg/mL; Invivogen, San Diego, USA). The granulosa cells were finally cultured in an incubator at 37 °C and containing 5% CO_2_. In this study, the experimental protocols were reviewed and approved by the Huazhong Agriculture University Institutional Committee on Animal Care and Use. For cell treatment, exosomes at a concentration of 100 µg/mL were added after the granulosa cells and reached 70–80% confluency. The granulosa cells and culture medium were collected for further research.

### 4.10. Detection of Expression of miRNAs and Genes by Real-Time PCR

Granulosa cells were harvested after treatment for 24 h with cow-milk-derived exosomes. The total RNA was extracted using an RNAprep Pure Cell Kit (Tiangen, Beijing, China), and then the first strand cDNA was synthesized with oligo (dT) using a cDNA Synthesis Kit (Thermo Scientific, Waltham, MA, USA) with DNaseI. Briefly, quantitative real-time PCR was performed on a LightCycler 480 II Real-Time PCR System (Roche, Penzberg, Germany) containing LightCycler 480 SYBR Green I Master Mix (5 μL), specific primer (0.5 μM for each primer), reverse transcribed cDNA (1 μL), and RNase and DNase-free ddH_2_O (3 μL). Amplification was performed as follows: 95 °C for 5 min, 40 cycles at 95 °C for 20 s, corresponding annealing temperatures for 20 s, 72 °C for 20 s; and a melting curve analysis was performed from 65 °C to 95 °C to confirm specific PCR products. The milk-derived exosome miRNAs and granulosa cell genes were detected according to the protocol of the miRcute Plus miRNA First Strand cDNA Kit and qPCR Kit (Tiangen, Beijing, China). The primers designed for detecting mRNA and miRNAs are listed in Table 1. Finally, the relative expression of mRNAs and miRNA was normalized to β-actin or U6 levels, and the expression levels were analyzed using the 2^−∆∆CT^ method [92].

### 4.11. Endocrine Secretion Detection

The hormone level was assessed in granulosa cells treated with exosomes derived from milk. The culture medium was collected 24 h after the granulosa cell treatment with exosomes. The cell culture medium was collected and centrifuged at 1000× *g* for 15 min. Finally, the culture medium was frozen at −80 °C until use. The measurement of progesterone and estradiol were carried out according to the manufacturer’s protocols of the bovine enzyme-linked immunosorbent assay (ELISA) kits (Shanghai Bogoo Biological Technology Co., Ltd., Shanghai, China). The sensitivity of estradiol was 1.0 pg/mL, and that of progesterone was 0.1 ng/mL.

### 4.12. Statistical Analysis

The data are presented as the mean ± standard deviation (SD) of three replicates. Significant differences were determined using one-way ANOVA with SPSS V17, and treatment means were compared using Tukey’s test for post-hoc multiple comparisons in SPSS V17 software. A t-test was used to determine the significance of differences between two groups. *p* < 0.05 was considered to indicate a significant difference.

## 5. Conclusions

This study first demonstrated the character of exosomes derived from estrous and non-estrous cow milk and the differentially expressed exosomal miRNAs. The results indicated that cow estrus could affect the milk exosome secretion and the expression of exosomal miRNAs, and the differentially expressed exosomal miRNAs were involved in reproduction and steroid hormone biosynthesis signaling pathways. Importantly, estrous and non-estrous cow-milk-derived exosomes both could promote the secretion of estradiol and progesterone in bovine granulosa cells. In addition, we found that milk exosomes could inhibit bovine granulosa cell apoptosis-related gene expression. This basic research also provides a basis for further investigating milk-derived exosomes and exosomal miRNA effects on ovary function and reproduction. However, more research is required to explore the role of exosomes in improving reproductive efficiency in an in vivo model.

## Figures and Tables

**Table 1:** Sequences of primer pairs for quantitative real-time PCR.

Gene	Forward Primer Sequence (5′→3′)	Reverse Primer Sequence (5′→3′)
CYP11A1	ATGCTGGAGGAGACAGTGAACC	GCAGTAGAGGATGCCTGGGTAA
CYP19A1	CACCCATCTTTGCCAGGTAGTC	ACCCACAGGAGGTAAGCCTATAAA
StAR	GTG GAT TTT GCC AAT CAC CT	TTATTG AAA ACG TGC CAC CA
RUNX2	AAGGCAAGGCTAGGTGGAAT	AGAGGGGCACAGACTTTGAA
HSD-3β	TGCCACAATCTGACCGCATC	CTCCACCAACAGGCAGATGA
Bcl-2	CGCATCGTGGCCTTCTTTGAGTT	GCCGGTTCAGGTACTCAGTCAT
p53	CCTCCCAGAAGACCTACCCT	CTCCGTCATGTGCTCCAACT
Caspase-3	CAGACAGTGGTGCTGAGGATGA	GCTACCTTTCGGTTAACCCGA
β-actin	CATCGGCAATGAGCGGTTCC	CCGTGTTGGCGTAGAGGTCC
bta-miR-3141	AACAATGAGGGCGGGTGGA	
bta-miR-154a	ACCACCGTAGGTTATCCGTGT	
bta-miR-432	AACCGGTCTTGGAGTAGGTCA	
bta-miR-339b	AACAAGTCCCTGTCCTCCAGG	
bta-let-7a-3p	CTATACAATCTACTGTCTTTC	
bta-miR-1777a	ATTAATTGGGGGCGGTGGG	
bta-miR-29c	GGGTAGCACCATTTGAAAT	
bta-miR-502b	AACCATGAATCCACCTGGGC	
bta-miR-664a	AACGATACAGGCTGGGGTGT	
bta-miR-409b	AACAATGGGGTTCACCGAGC	
U6	GCTTCGGCAGCACATATACTAAAAT	

## Data Availability

Not applicable.

## Supplementary Materials

The supporting information can be downloaded at: https://www.mdpi.com/article/10.3390/ijms24119608/s1.
